# Pathogenicity of *Brucella* sp. ST27 *Kogia sima* Isolates in Murine and Cell Models

**DOI:** 10.3390/tropicalmed11040098

**Published:** 2026-04-07

**Authors:** Andrea Romero-Magaña, Carlos Chacón-Díaz, Alejandro Alfaro-Alarcón, Marcela Suárez-Esquivel, Esteban Chaves-Olarte, Gabriela Hernández-Mora, Edgardo Moreno, Elías Barquero-Calvo

**Affiliations:** 1 Programa de Investigación en Enfermedades Tropicales, Escuela de Medicina Veterinaria, Universidad Nacional, Heredia 40101, Costa Rica; romerolucia0105@gmail.com (A.R.-M.); marcela.suarez.esquivel@una.ac.cr (M.S.-E.); edgardo.moreno.robles@una.ac.cr (E.M.); 2 Laboratorio de Patología, Escuela de Medicina Veterinaria, Universidad Nacional, Heredia 40101, Costa Rica; carlos.chacondiaz@ucr.ac.cr (C.C.-D.); esteban.chaves@ucr.ac.cr (E.C.-O.); 3 Institute of Virology, Berlin Institute of Health, Charité-Universitätsmedizin Berlin, Corporate Member of Freie Universität Berlin and Humboldt-Universität zu Berlin, 10117 Berlin, Germany; alejandro.alfaro.alarcon@una.ac.cr; 4 Centro de Investigación en Enfermedades Tropicales, Facultad de Microbiología, Universidad de Costa Rica, San José 2060, Costa Rica; 5 Servicio Nacional de Salud Animal, Heredia 11401, Costa Rica; gabriela.hernandez.m@senasa.go.cr

**Keywords:** *Brucella* sp. ST27, marine mammals, zoonotic potential, pathogenicity, intracellular replication, mice

## Abstract

Members of the genus *Brucella* are bacterial pathogens of global importance, and their increasing detection in marine mammals has raised concerns for wildlife conservation and public health. In this study, we evaluated the biological and pathogenic characteristics of two *Brucella* sp. sequence type 27 (ST27) isolates obtained from a dwarf sperm whale (*Kogia sima*). We compared them with terrestrial and marine *Brucella* reference strains. We assessed resistance to polymyxin B and human serum complement, intracellular infection dynamics in HeLa epithelial cells, persistence in a murine model, and associated hematological and histopathological changes, and analyzed lipopolysaccharide (LPS) profiles. The *Kogia* isolates exhibited resistance to polymyxin B and serum complement, comparable to that of *B. abortus* 2308W and marine mammal *Brucella* strains. In HeLa cells, the isolates displayed distinct, strain-specific intracellular infection dynamics. In the murine model, both isolates persisted in the spleen and induced granulomatous lesions. However, splenic bacterial loads and histopathological scores were generally lower than those observed with *B. abortus* 2308W, which exhibited the highest virulence among the strains evaluated. Hematological alterations associated with *Kogia* isolates were also less pronounced than those induced by *B. abortus* 2308W, indicating an intermediate and strain-dependent virulence phenotype without evidence of enhanced virulence relative to the terrestrial reference strain. Western blot analyses showed that *Brucella* sp. ST27 isolates were not recognized by anti-*B. abortus* or anti-O-antigen monoclonal antibodies, while exhibiting a distinct recognition pattern with anti-*B. canis* serum, indicating differences in surface antigen composition. Comparative whole-genome analysis identified a limited number of isolate-specific variants affecting coding and intergenic regions. Collectively, these findings highlight phenotypic and genetic features of *Brucella* sp. ST27 from *Kogia sima*, which distinguishes it from other marine and terrestrial *Brucella* strains and supports further investigation into its biological behavior and potential public health relevance.

## 1. Introduction

Members of the genus *Brucella* are bacterial pathogens of mammals with a global distribution, responsible for significant economic losses and considerable human morbidity. Several species of this genus, including *Brucella melitensis*, *Brucella abortus*, and *Brucella suis*, are facultative extracellular–intracellular pathogens that can replicate extensively in the mononuclear phagocyte system and the reproductive system. These organisms can survive and replicate within both nonphagocytic and phagocytic cells, a feature that contributes to the establishment of chronic infections in mammalian hosts [1,2,3,4].

In recent years, the presence of *Brucella* spp. in marine mammals has received increasing attention due to its potential implications for wildlife conservation and public health [5]. Since the first isolation of *Brucella* spp. from marine mammals in 1994 [6,7], numerous cases have been reported worldwide. To date, two *Brucella* species have been formally described in marine mammals: *B. ceti*, which primarily infects cetaceans, and *B. pinnipedialis*, which predominantly affects pinnipeds [8]. Infections caused by *B. ceti* have been associated with a wide range of pathological conditions, including meningoencephalitis, discospondylitis, subcutaneous abscesses, endometritis, and myocarditis, frequently leading to stranding events and, in some cases, fatal outcomes. In contrast, detailed pathological descriptions of *B. pinnipedialis* infections remain comparatively scarce [5,9,10].

Beyond their impact on marine mammals, marine mammal *Brucella* spp. have also been implicated in human infections. To date, three naturally acquired human cases and one laboratory-acquired infection involving marine mammal *Brucella* strains have been documented [11,12,13]. Subsequent multilocus sequence typing (MLST) analyses demonstrated that the naturally infected individuals harbored strains with a unique genotype, designated sequence type 27 (ST27) [14,15]. Notably, this genotype has also been isolated from marine mammals presenting fetal pneumonia and abortion-associated lesions [6,16,17,18,19]. As a result, *Brucella* sp. ST27 has attracted particular interest due to its zoonotic potential and its apparent capacity to infect both marine mammals and humans [15].

The increasing detection of *B. ceti* and *B. pinnipedialis* in marine mammals, along with their expanding host range and geographic distribution, has raised concerns about their zoonotic potential, particularly for *Brucella* sp. ST27. Possible routes of transmission to humans, including direct contact with infected animals or exposure through the food chain, represent emerging public health challenges. However, despite growing epidemiological and molecular evidence, the pathogenic potential of ST27 strains and their biological behavior across different experimental models remain incompletely understood.

In this context and building on clinical observations from naturally infected marine mammals, experimental animal models of brucellosis, and in vitro infection systems, the present study aims to contribute to a better understanding of the pathogenic characteristics of *Brucella* sp. ST27 isolated from a *Kogia sima* specimen [18]. Specifically, we evaluated phenotypic traits, intracellular infection dynamics, host responses, and genomic variation, and compared these features with those of other marine mammal *Brucella* species and the classical terrestrial reference strain *B. abortus* 2308W.

## 2. Materials and Methods

### 2.1. Bacterial Strains and Growth Conditions

All bacterial strains used in this study are listed in Table 1. Bacteria were cultured as previously described [20,21,22]. *Brucella* sp. sequence type 27 (ST27) isolates bmarCR39b and bmarCR42b, obtained from a *Kogia sima* specimen [18], were grown and maintained in standard trypticase soy broth (TSB) or trypticase soy agar (TSA) (BD, Sparks, MD, USA) at 37 °C under 5% CO_2_. Bacterial stocks were stored at −80 °C in skim milk (BD, Sparks, MD, USA) supplemented with 20% glycerol (Sigma-Aldrich, St. Louis, MO, USA). In selected assays, *B. abortus* 2.13 (bvrS mutant) and *B. canis* bcanCR12 were included as control strains.

### 2.2. Determination of the Minimum Inhibitory Concentration to Polymyxin B

To determine the susceptibility of *Brucella* strains to polymyxin B, bacteria were cultured overnight in trypticase soy broth (TSB) at 37 °C with shaking at 200 rpm. Bacterial suspensions were adjusted to a 1 McFarland standard in sterile saline solution (0.85%) and subsequently diluted 1:100 in TSB. Polymyxin B sulfate (Sigma-Aldrich, St. Louis, MO, USA) was dissolved in sterile water to obtain a stock solution of 500 µg/mL, and two-fold serial dilutions were prepared in TSB in 96-well microtiter plates (100 µL per well). An equal volume (100 µL) of the bacterial suspension was added to each well, and assays were performed in triplicate. Plates were incubated at 37 °C for 48 h. Following incubation, bacterial growth was evaluated visually, and the minimum inhibitory concentration (MIC) was defined as the lowest concentration of polymyxin B that completely inhibited visible growth, as previously described [26]. MIC values are expressed as the mean ± standard deviation from at least three independent experiments.

### 2.3. Serum Bactericidal Activity

The sensitivity of *Brucella* strains to the bactericidal activity of normal serum was evaluated as previously described, with minor modifications [27]. Briefly, late-exponential-phase bacteria were adjusted to a final concentration of approximately 10^4^ colony-forming units (CFU)/mL in phosphate-buffered saline (PBS). Aliquots of 200 µL of the bacterial suspension were mixed with 400 µL of non-immune human serum in sterile microcentrifuge tubes. As a control, complement-inactivated human serum was prepared by adding dehydrated yeast (*Saccharomyces cerevisiae*) and incubating at 56 °C for 45 min. The treated serum was subsequently centrifuged at 5000 rpm for 5 min to remove particulate material. Bacterial suspensions were incubated at 37 °C for 90 min. After incubation, 80 µL of each sample was plated in triplicate onto trypticase soy agar (TSA) plates using the Drigalsky spread plate method. Plates were incubated at 37 °C, and viable bacteria were quantified as CFU. Results are expressed as the mean percentage of CFU survival ± standard deviation from one representative experiment performed in triplicate, with at least three independent assays.

### 2.4. Crystal Violet Staining and Acriflavine Agglutination Tests for Differentiation of Smooth and Rough Brucella Isolates

Differentiation between smooth and rough *Brucella* phenotypes was performed using crystal violet staining and the acriflavine agglutination test, following the procedures described by Alton et al. [28]. Briefly, isolates were grown on trypticase soy agar (TSA) at 37 °C under 5% CO_2_ for 3–4 days. For crystal violet staining, individual colonies were flooded with a 1:40 dilution of the dye for approximately 15 s. Smooth colonies excluded the stain and appeared pale or translucent, whereas rough colonies retained the dye and appeared deep violet. In parallel, the acriflavine agglutination test was performed by mixing fresh bacterial growth with 0.1% acriflavine solution on a glass slide; rough strains exhibited rapid visible agglutination, while smooth strains remained uniformly suspended.

### 2.5. Lipopolysaccharide (LPS) Characterization by Western-Blot

*Brucella* whole-cell extracts, from a panel of selected strains, were subjected to SDS-PAGE (10%) and transferred to a PVDF membrane. Recognition patterns for the selected panel of strains were compared using specific antibodies against *Brucella* species, including (A) polyclonal anti-*B. canis*, (B) polyclonal anti-*B. abortus*, and (C) polyclonal anti-*Brucella* sp. (*Kogia*), which were used at a 1:500 dilution, and (D) monoclonal antibody M84, used to determine the presence of O-polysaccharide, which was used at a 1:250 dilution. All antibodies were diluted in PBS-1% nonfat milk-2% Tween 20. A secondary anti-mouse IgG-HRP at a 1:1000 dilution was used for detection. Image capturing was performed using a ChemiDoc MP imaging system (Bio-Rad Laboratories, Hercules, CA, USA).

### 2.6. Murine Experiments

Animals were provided by the Servicio Nacional de Salud Animal (SENASA), Ministry of Agriculture (MAG), Costa Rica, and housed at the animal research facility of the Veterinary School, Universidad Nacional, Costa Rica, until use. Mice were maintained under biosafety containment conditions, with food and water available ad libitum, and under a 12 h light/dark cycle. Eight-week-old female and male CD-1 mice (18–22 g) were randomly assigned to five experimental groups (*n* = 12 per group). Mice were infected with *B. abortus* 2308W, *B. ceti* B14/94, *B. pinnipedialis* B2/94, *Brucella* sp. bmarCR39b, or *Brucella* sp. bmarCR42b. Each mouse was inoculated intraperitoneally with 0.1 mL of sterile phosphate-buffered saline (PBS) containing 10^6^ colony-forming units (CFU) of the corresponding strain, as determined by serial dilution and plate counting. At 8 and 30 days post-infection, spleen and blood samples were collected. Splenic bacterial loads were determined and expressed as CFU/g of tissue, as previously described [29].

### 2.7. Histopathology and Hematology Analyses

For histopathological analyses, spleens collected at 8 and 30 days post-infection were fixed in 10% neutral buffered formalin, routinely processed, and stained with hematoxylin and eosin, as previously described [30]. Splenic inflammatory changes were evaluated using a semi-quantitative scoring system and graded on a scale from 0 (no inflammatory changes) to 4 (severe inflammation), according to established criteria [31,32,33]. Hematological analyses were performed using a VetScan HM5 Hematology Analyzer (Abaxis, Inc., Union City, CA, USA) according to the manufacturer’s instructions.

### 2.8. Cell Culture and Intracellular Replication Quantification

Human cervical carcinoma cells (HeLa; ATCC CCL-2) were maintained in Dulbecco’s Modified Eagle Medium (DMEM) supplemented with 5% fetal bovine serum, 2.5% sodium bicarbonate, and 1% glutamine at 37 °C under 5% CO_2_. Two days prior to infection, cells were seeded at a density of 5 × 10^5^ cells per well in 24-well tissue culture plates. *Brucella* strains were grown to the late exponential phase and diluted in DMEM to achieve a multiplicity of infection (MOI) of 500. Plates were centrifuged at room temperature for 5 min at 1500 rpm to promote bacterial contact and incubated for 45 min at 37 °C under 5% CO_2_. Cells were then washed three times with phosphate-buffered saline (PBS) and incubated for 1 h in DMEM containing 100 µg/mL gentamicin to eliminate extracellular bacteria. Subsequently, cells were maintained in DMEM supplemented with 5 µg/mL gentamicin. At 0, 4, 24, and 48 h post-infection, wells were washed twice with PBS and lysed with 0.5 mL sterile water for 10 min. Intracellular bacteria were quantified by serial dilution and plating on trypticase soy agar (TSA), followed by incubation at 37 °C under 5% CO_2_ for CFU determination, as previously described [34].

### 2.9. Whole Genome Sequence Comparison

Raw sequencing reads from *Brucella* sp. isolates bmarCR39b and bmarCR42b, previously published [18] under accession numbers ERR3799635 and ERR3799636, were analyzed to identify genome-wide variations. Reads were mapped against the reference genome of *Brucella suis* biovar 1 strain 1330 (GCA_000007505) using Snippy v4.6.0 [35], which integrates BWA-MEM for read alignment and FreeBayes for variant calling. Default parameters were applied, including a minimum mapping quality of 60 and a minimum read depth of 10. Variant confidence was assessed using standard VCF metrics, including total read depth (DP), alternate allele read count (AO), reference allele reads (RO), and Phred-scaled quality score (QUAL). Only homozygous variants (genotype 1/1) with strong alternate allele support (AO ≥ 10, RO = 0, QUAL > 200) were classified as high-confidence and retained for downstream interpretation. Complete variant statistics and filtering criteria are provided in Supplementary Dataset S1. For comparative purposes, a de novo genome assembly of bmarCR42b was generated using Unicycler v0.4.8 [36] with Illumina-only data, and assembly quality was evaluated using QUAST v5.0.2 [37,38,39]. However, due to the high assembly fragmentation and limited core-genome overlap among isolates, read mapping to a typical reference genome was selected as the primary approach for robust, consistent variant detection.

### 2.10. Statistics

Statistical analyses were performed using GraphPad Prism software (version 10.0.2; GraphPad Software, LLC, San Diego, CA, USA) and R software (version 4.3.1). Unless otherwise stated, data are presented as mean ± standard deviation (SD) from at least three independent experiments. For comparisons involving multiple groups, one-way analysis of variance (ANOVA) was used when data were normally distributed, whereas the nonparametric Kruskal–Wallis test was used when normality assumptions were not met. Post hoc multiple-comparison tests were performed as appropriate. Differences were considered statistically significant at *p* < 0.05, with additional significance thresholds indicated where relevant (*p* < 0.01, *p* < 0.001, and *p* < 0.0001).

## 3. Results

### 3.1. Resistance to Serum Complement and Polymyxin B

**Figure 1:**
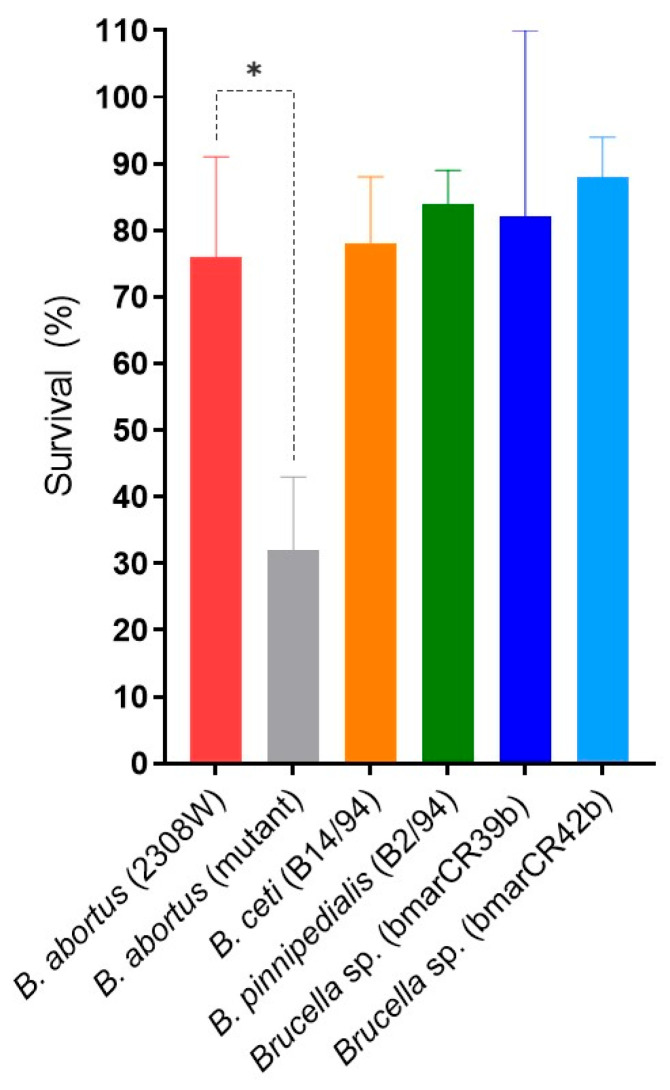
Resistance of *Brucella* strains to non-immune human serum. Survival percentage of *Brucella* strains after incubation in non-immune human serum. Bacterial suspensions were incubated with non-immune human serum for 90 min at 37 °C, and survival was determined by colony-forming unit (CFU) count. Results are expressed as mean ± standard deviation (SD) of the percentage of surviving bacteria from one representative experiment performed in triplicate, with at least three independent assays. Significant differences relative to the *B. abortus* 2308W reference strain are indicated (* *p* ≤ 0.05).

Given the well-established association between pathogenic *Brucellaceae* species and resistance to the bactericidal activity of serum complement and polycationic peptides [40], we conducted a series of sensitivity assays to evaluate the susceptibility of *Brucella* strains to human serum and the antibiotic polymyxin B. In the serum complement assay, no significant differences in survival percentages were observed between the *B. abortus* 2308W reference strain and the *Kogia sima* isolates (bmarCR39b and bmarCR42b) (Figure 1), with comparable survival levels detected across these strains. Similarly, *B. ceti* B14/94 and *B. pinnipedialis* B2/94 exhibited high survival percentages following exposure to serum complement. As a control, the *B. abortus* 2.13 strain (bvrS mutant), previously described as attenuated [25], was included and exhibited a minor survival rate in the presence of serum complement.

**Figure 2:**
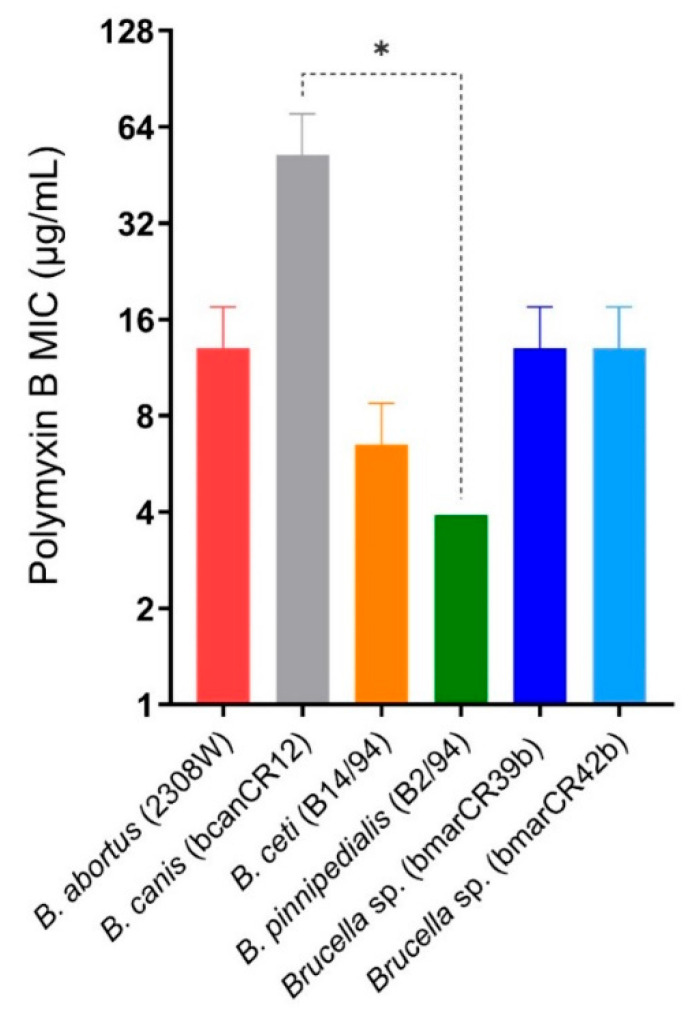
Susceptibility of *Brucella* strains to polymyxin B. Minimal inhibitory concentrations (MICs) of polymyxin B against different *Brucella* strains. Bacteria were exposed to two-fold serial dilutions of polymyxin B for 48 h at 37 °C, and MICs were determined as the lowest concentration preventing visible growth. Data represent mean ± SD of MIC values obtained from three independent experiments performed in duplicate. Significant differences are indicated (* *p* ≤ 0.05).

Exposure to increasing concentrations of polymyxin B did not reveal significant differences between the *K. sima* isolates and the *B. abortus* 2308W reference strain (Figure 2). In contrast, *B. ceti* B14/94 and *B. pinnipedialis* B2/94 displayed lower minimum inhibitory concentrations (MICs) than *B. abortus* 2308W, with MICs of 6.5 µg/mL and 3.9 µg/mL, respectively; however, no significant differences were observed between these two marine strains. The *B. canis* strain (bcanCR12), included as a control [24], was resistant to polymyxin B and showed significant differences when compared to the *B. pinnipedialis* B2/94 strain. Overall, these assays revealed comparable susceptibility profiles to serum complement and polymyxin B among marine *Brucella* strains and the *B. abortus* 2308W reference strain, while preserving species- and strain-level differences detectable under specific experimental conditions.

### 3.2. Crystal Violet Staining, Acriflavine Agglutination Tests and Western Blot LPS Banding Patterns

**Figure 3:**
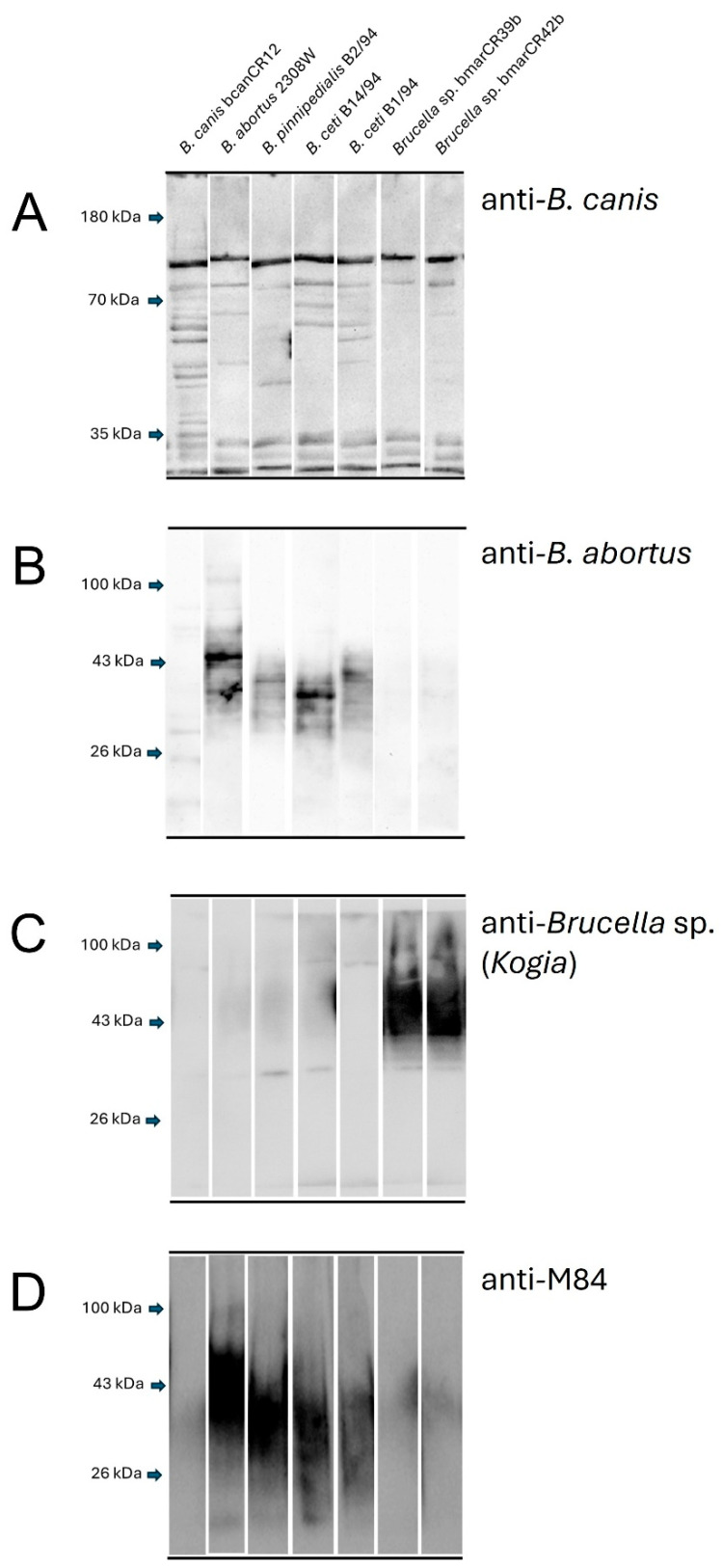
Lipopolysaccharide (LPS) Western blot analysis. Whole-cell lysates of *Brucella* strains were analyzed by Western blot using (**A**) polyclonal anti-*Brucella canis* antibody, (**B**) polyclonal anti-*Brucella abortus* antibody, (**C**) polyclonal anti-*Brucella* sp. (*Kogia*) antibody, and (**D**) monoclonal anti-O-antigen antibody M84.

The isolates bmarCR39b and bmarCR42b were identified as smooth phenotypes based on crystal violet exclusion and the absence of agglutination in the acriflavine test. These characteristics were comparable to those observed for smooth reference strains. Western blot analysis using a polyclonal anti-*Brucella canis* antibody revealed a similar recognition pattern for the *Kogia sima* isolates bmarCR39b and bmarCR42b, which was clearly distinct from the patterns observed for the other *Brucella* reference strains included in the analysis (Figure 3A). When membranes were probed with a polyclonal anti-*Brucella abortus* antibody, strong reactivity was observed for smooth reference strains. In contrast, the *Kogia sima* isolates exhibited minimal to no reactivity, with only faint or diffuse signals detected under these conditions (Figure 3B). Probing with a polyclonal anti-*Brucella* sp. (*Kogia*) antibody resulted in specific recognition of the *Kogia sima* isolates. At the same time, no detectable signal was observed for the other marine or terrestrial reference strains analyzed (Figure 3C). Finally, when membranes were probed with the monoclonal anti-O-antigen antibody M84, clear reactivity was detected in the marine reference strains *B. ceti* B14/94, *B. ceti* B1/94, and *B. pinnipedialis* B2/94; however, no signal was observed for the *Kogia sima* isolates (Figure 3D).

### 3.3. Brucella Persistence in the Mouse Spleen

**Figure 4:**
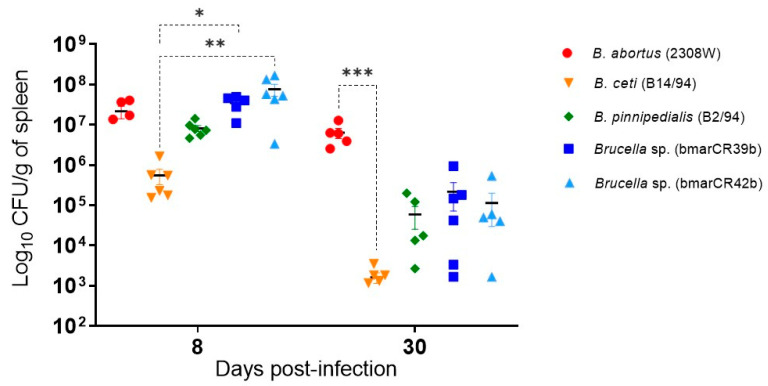
*Brucella* persistence in the murine spleen. Bacterial loads in the spleen of CD-1 mice infected intraperitoneally with 10^6^ CFU of *Brucella* strains. Mice were euthanized at 8 and 30 days post-infection, and spleen bacterial burdens were determined by CFU enumeration. Results are expressed as mean ± standard error of the mean (SEM) of log_10_ CFU per spleen. Statistical significance is indicated as * *p* ≤ 0.05, ** *p* ≤ 0.01, and *** *p* ≤ 0.001.

The murine model has been extensively used to study brucellosis, and the ability of *Brucella* strains to persist in the spleen has commonly been used as a parameter for comparative assessment of infection dynamics in vivo [41]. Accordingly, splenic bacterial loads of *Brucella* sp. *Kogia sima* were quantified in infected mice at 8 days post-infection (p.i.), during the acute phase, and at 30 days p.i., during the chronic phase. At day 8 p.i., splenic bacterial loads of the *K. sima* isolates were comparable to those detected in mice infected with *B. abortus* 2308W (Figure 4). In contrast, *B. ceti* B14/94 exhibited lower bacterial loads at this point, with a statistically significant difference when compared to both *K. sima* isolates. At day 30 p.i., a decline in splenic bacterial loads was observed for all marine mammal *Brucella* strains when compared to *B. abortus* 2308W, with *B. abortus* 2308W consistently exhibiting the highest splenic bacterial burdens among the strains evaluated at this time point. This reduction was particularly evident for *B. ceti* B14/94, which showed significantly lower bacterial loads than those observed in mice infected with *B. abortus* 2308W at this later time point.

### 3.4. Hematological and Histopathological Profiles

We also evaluated whether bacterial infection in mice affected hematological parameters or altered splenic histopathological lesions. Hematological analyses performed at eight days post-infection (p.i.) (Table 2) showed that mice infected with *B. ceti* B14/94 and *B. pinnipedialis* B2/94 exhibited significant differences in lymphocyte and neutrophil percentages, as well as in platelet counts, compared to mice infected with *B. abortus* 2308W. In contrast, no significant differences were observed between mice infected with the *Kogia sima* isolates and those infected with *B. abortus* 2308W at this time point. Overall, the hematological profiles associated with *K. sima* isolates were highly similar to those observed for *B. ceti* B14/94 and *B. pinnipedialis* B2/94. At 30 days post-infection, significant differences in specific hematological parameters were observed between mice infected with marine mammal *Brucella* strains and those infected with *B. abortus* 2308W, particularly in selected lymphocyte and neutrophil values, as detailed in Table 3. These differences were consistent with the reduced splenic bacterial loads detected for marine strains at this time point. Among the strains evaluated, *B. ceti* B14/94 displayed the most significant number of hematological differences relative to *B. abortus* 2308W at both 8 and 30 days p.i.

**Figure 5:**
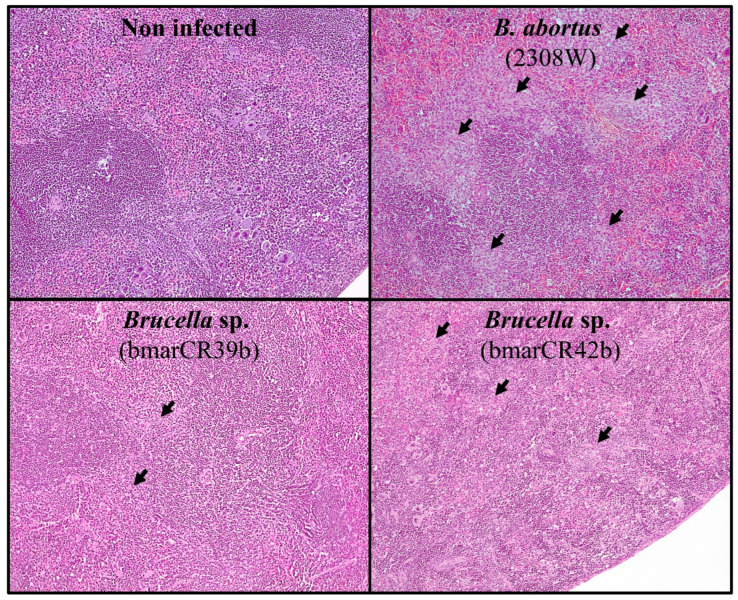
Histopathological changes in the spleens of mice infected with *Brucella* sp. from *Kogia sima*. Representative histological sections of spleens from CD-1 mice infected intraperitoneally with 10^6^ CFU of *Brucella* strains and euthanized at 8 days post-infection. Sections were stained with hematoxylin and eosin. Arrows indicate the presence of granulomatous inflammatory lesions.

**Figure 6:**
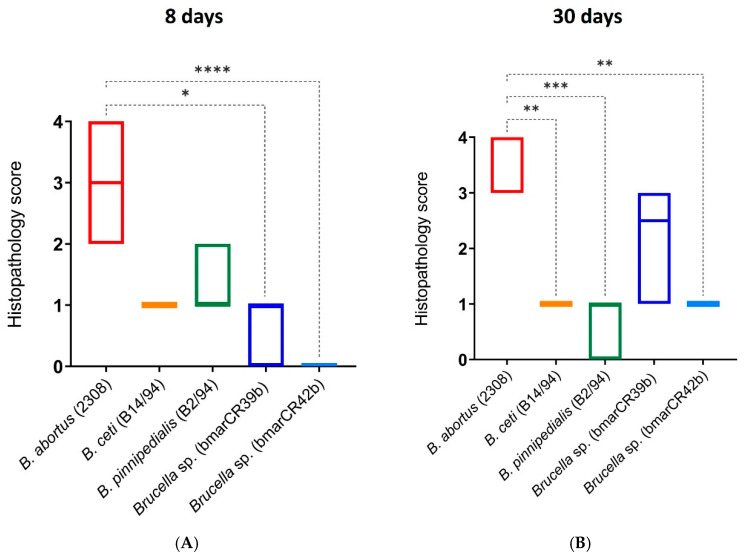
Granulomatous inflammation score in the murine spleen. Semi-quantitative scoring of granulomatous inflammation in spleens of CD-1 mice infected intraperitoneally with 10^6^ CFU of *Brucella* strains. Spleens were collected at (**A**) 8 and (**B**) 30 days post-infection and scored on a scale from 0 (no inflammation) to 4 (severe inflammation). Data are presented as median with minimum and maximum values. Statistical significance is indicated as * *p* ≤ 0.05, ** *p* ≤ 0.01, *** *p* ≤ 0.001, and **** *p* ≤ 0.0001.

Histopathological examination of spleen tissues showed that infection with all *Brucella* strains induced lesions characteristic of murine brucellosis, including granuloma formation [41,42]. However, when compared to *B. abortus* 2308W, splenic lesions were generally less conspicuous in mice infected with *Brucella* sp. *K. sima* isolates (Figure 5). Granulomatous inflammation was semi-quantitatively scored on a scale from 0 (absence of inflammation) to 4 (severe inflammation) for all strains at 8 and 30 days p.i. At 8 days p.i., significant differences in histopathological scores were observed between *B. abortus* 2308W and *Brucella* sp. *K. sima* isolates, with lower scores detected in the latter group (Figure 6), supporting an intermediate and strain-dependent virulence phenotype for the *K. sima* isolates in the murine model. This difference was observed despite comparable splenic bacterial loads at this time point. Mice infected with *B. ceti* B14/94 and *B. pinnipedialis* B2/94 exhibited granulomatous inflammatory responses similar to those observed for *K. sima* isolates; however, these differences did not reach statistical significance. A comparable pattern was observed at 30 days p.i. Significant differences in granulomatous inflammation scores were detected between *B. abortus* 2308W and *B. ceti* B14/94, *B. pinnipedialis* B2/94, and *Brucella* sp. bmarCR42b strains (Figure 6). No statistically significant difference was observed for the *Brucella* sp. bmarCR39b isolate at this time point, indicating strain-specific variation in the severity of granulomatous inflammatory responses among *K. sima* isolates.

### 3.5. Infection Dynamics in HeLa Cells

**Figure 7:**
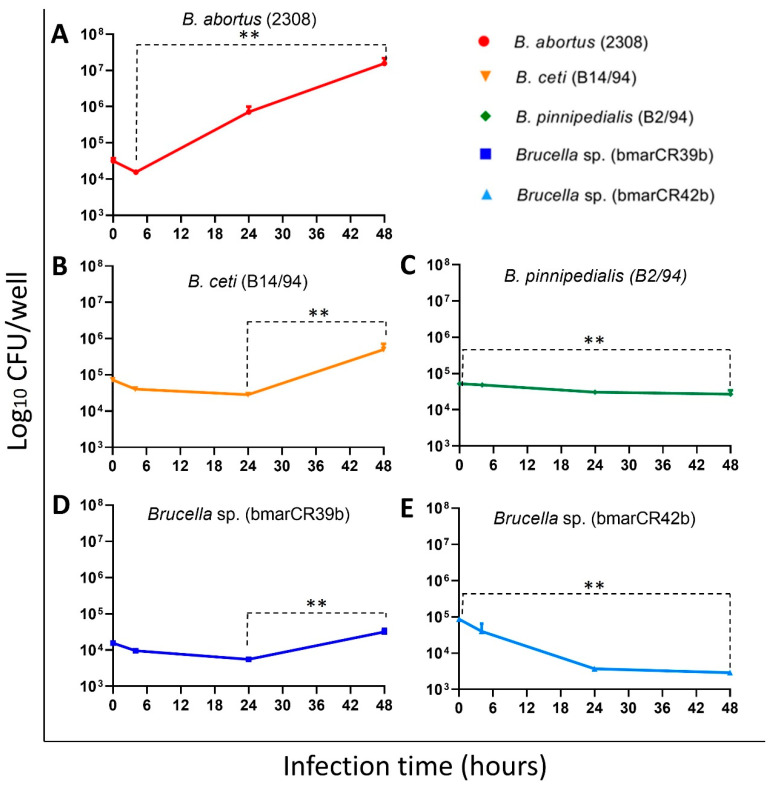
Infection dynamics of *Brucella* strains in HeLa cells. HeLa epithelial cells were infected with *Brucella* strains at a multiplicity of infection (MOI) of 500 using a gentamicin protection assay. Intracellular viable bacteria were quantified at the indicated time points by CFU enumeration. Panels (**A**–**E**) correspond to individual *Brucella* strains. Results are expressed as mean log CFU per well ± SD from one representative experiment performed in duplicate and representative of three independent experiments. Significant differences between 0 and 48 h post-infection are indicated (** *p* ≤ 0.01).

Next, we evaluated the ability of the different *Brucella* strains to infect and replicate intracellularly in HeLa epithelial cells, a commonly used in vitro model to assess intracellular survival and replication of *Brucella* spp. [43]. Distinct intracellular infection patterns were observed among the *Kogia sima* isolates. Following infection, both *K. sima* isolates initially reached comparable intracellular bacterial loads, with approximately 4.2 log CFU detected at early time points (Figure 7D,E). At 48 h post-infection (p.i.), divergent intracellular dynamics were observed between the two isolates. The *Brucella* sp. bmarCR39b strain exhibited a significant increase in intracellular bacterial numbers, reaching approximately 4.5 log CFU. In contrast, the *Brucella* sp. bmarCR42b strain showed a significant reduction in intracellular CFU of approximately one log at the same time point. These contrasting patterns reflect isolate-specific differences in intracellular infection dynamics within epithelial cells. As expected, the *B. abortus* 2308W reference strain displayed robust intracellular replication in HeLa cells, with a marked increase in intracellular bacterial load at 48 h p.i. (Figure 7A). The marine mammal strain *B. ceti* B14/94 also demonstrated the ability to infect and replicate within HeLa cells, showing an increase in intracellular CFU at 48 h p.i., although to a lesser extent than *B. abortus* 2308W (Figure 7B). In contrast, *B. pinnipedialis* B2/94 exhibited limited intracellular replication in this epithelial cell model (Figure 7C). Following an initial intracellular bacterial load of approximately 4.4 log CFU, a reduction of roughly 0.5 log CFU was observed at 48 h p.i., indicating restricted intracellular persistence under these experimental conditions.

### 3.6. Whole-Genome Variant Detection

To assess genome-wide variation between the two *Brucella* sp. *Kogia sima* isolates, short-read mapping was performed using the *B. suis* 1330 genome as a standard reference. This strategy was selected over a direct genome-to-genome comparison because a de novo assembly of ERR3799636 generated with Unicycler from Illumina-only data resulted in a highly fragmented genome (79 contigs), limiting core genome overlap and reliable detection of isolate-specific variants (assembly summary statistics are provided in Supplementary Dataset S1). Mapping both datasets to a shared reference genome enabled consistent variant calling across isolates. Variant calling revealed isolate-specific genetic variation affecting both coding sequences and intergenic regions, including single-nucleotide polymorphisms (SNPs), small insertions and deletions (indels), and complex mutations (Supplementary Dataset S1). For isolate bmarCR39b (ERR3799635), a total of 12 variant sites were identified relative to the reference genome, of which 7 met the high-confidence filtering criteria. These high-confidence variants included 2 SNPs, 2 small insertions, and 3 deletions. Among coding-region variants, a missense SNP (p.Val35Met) was detected in the ISBm1 transposase orfA gene, and a frameshift deletion (p.Tyr330fs) was identified in a Gfo/Idh/MocA family oxidoreductase (BS1330_II0400). Additional variants were located in genes encoding hypothetical proteins and in intergenic regions. For isolate bmarCR42b (ERR3799636), 12 isolate-specific variants were also detected, of which 9 were classified as high confidence. These included several SNPs and complex indels affecting both coding and non-coding regions. Notable coding-region variants comprised an in-frame insertion in the xanthine dehydrogenase gene (p.Thr352_Ala353insValSerAsnThr) and an in-frame deletion in a cobalamin synthesis–related protein (BS1330_II0979), resulting in the removal of 29 amino acids (p.His241_His269del). Additional variants were identified in genes encoding hypothetical proteins and in intergenic regions.

Collectively, these genomic differences indicate isolate-specific genetic divergence, with variation detected in genes associated with metabolic pathways, mobile genetic elements, and proteins of unknown function.

## 4. Discussion

We previously reported two *Brucella* strains (bmarCR39b and bmarCR42b) isolated from a stranded *Kogia sima* that are closely related to phylogenetic relatives of *Brucella* sp. sequence-type 27 (ST27) strains [18]. This genotype is of particular interest because all documented cases of naturally acquired human brucellosis presumably linked to the marine environment have been caused by ST27 strains [12,13]. To assess the pathogenic potential of these *K. sima* isolates, we evaluated a series of phenotypic and in vivo traits commonly used to characterize *Brucella* pathogenicity. We compared them with terrestrial and marine reference strains.

Our results show that bmarCR39b and bmarCR42b display several hallmark pathogenic traits of the family *Brucellaceae*, including resistance to polymyxin B and serum complement, a distinctive LPS banding pattern, intracellular survival in epithelial cells, bacterial persistence in the murine model, and induction of granulomatous inflammation in the spleen [1,41]. Together, these findings support the notion that ST27 isolates from *Kogia* possess core virulence-associated characteristics.

Importantly, these observations highlight a fundamental limitation of traditional experimental models. Marine *Brucella* strains, including *B. ceti* and ST27 isolates, are frequently recovered from stranded cetaceans exhibiting severe, often fatal disease, including neurological, cardiac, skeletal, and reproductive pathologies [8,44,45]. These clinical manifestations are rarely reproduced in murine models and differ markedly from the disease presentation in livestock species infected with terrestrial *Brucella* spp., where fetal loss is often the primary severe outcome [46]. Strikingly, many of the pathological features observed in cetaceans resemble those described in human brucellosis, reinforcing the zoonotic relevance of marine *Brucella* infections [15].

This mismatch between experimental virulence profiles and natural disease outcomes suggests that the reduced virulence observed in mice and cell lines reflects the limited ability of these models to replicate cetacean-specific host–pathogen interactions. For instance, *B. ceti* shows efficient intracellular replication in epithelial cells but limited persistence in mice, whereas ST27 strains display the opposite trend, despite both being associated with fatal infections in their natural hosts. These discrepancies highlight the limitations of conventional experimental systems in reproducing host-specific disease outcomes. Furthermore, ST27 strains isolated from *Kogia* are closely related to strains recovered from human brucellosis cases, underscoring their potential public health relevance [12,15].

The pathogenicity of *Brucella* species is closely linked to the composition and structure of the outer membrane, which influences internalization pathways, intracellular trafficking, and immune evasion [40]. In this study, both *K. sima* isolates exhibited resistance to serum complement, comparable to that of *B. abortus* 2308W and marine mammal reference strains. In contrast, their resistance to polymyxin B was more similar to *B. abortus* than to other marine *Brucella* isolates. Previous studies have shown that marine mammal-derived *Brucella* spp. display heterogeneous susceptibility to cationic peptides, suggesting strain-specific genomic or structural differences [47].

These resistance patterns suggest that bmarCR39b and bmarCR42b may share aspects of outer membrane composition with smooth terrestrial strains and particular marine *Brucella* species. Smooth *Brucella* strains possess a distinctive lipopolysaccharide (LPS) structure that contributes to resistance against antimicrobial peptides and complement activation [40,47,48,49]. Conversely, LPS-defective mutants exhibit increased susceptibility and attenuation [50]. Although the ST27 isolates were phenotypically classified as smooth based on crystal violet exclusion and negative acriflavine agglutination, their Western blot recognition profile differed from that of classical smooth marine and terrestrial *Brucella* strains. The absence of reactivity with the monoclonal anti-O-antigen antibody and anti-*B. abortus* serum, combined with partial recognition by anti-*B. canis* serum suggests structural differences in surface-exposed epitopes. These findings do not support a rough phenotype but rather indicate a distinct antigenic profile, potentially involving variation in O-polysaccharide structure or other outer membrane components. Further biochemical and structural analyses will be required to define the precise molecular basis of this differential antibody recognition.

Intracellular infection assays revealed differences in replication dynamics between the two *K. sima* isolates. While both strains survived within HeLa cells up to 48 h post-infection, bmarCR39b exhibited an intracellular replication profile resembling that of *B. ceti* B14/94, whereas bmarCR42b showed a more attenuated pattern similar to *B. pinnipedialis* B2/94. These phenotypic differences may be explained by strain-specific genomic variants affecting metabolic pathways, mobile genetic elements, cofactor biosynthesis, and hypothetical proteins with unknown functions. Notably, disruptive mutations identified in isolate bmarCR42b may influence gene stability or regulation.

Consistent with these phenotypic differences, genomic analysis revealed that although no disruptive mutations were identified in canonical virulence genes such as the *virB* operon or the *bvrR*/*bvrS* regulatory system, several isolate-specific variants were detected in genes encoding predicted outer membrane proteins, transporters, and regulatory elements. Importantly, no frameshift or nonsense mutations were observed in core smooth LPS biosynthesis genes, supporting the phenotypic classification of these isolates as smooth. Nevertheless, subtle amino acid substitutions in surface-associated or regulatory proteins may influence antigenic structure, immune recognition, or intracellular adaptation. These genomic differences could contribute to the distinct antibody recognition pattern observed in Western blot assays and to the strain-dependent virulence phenotypes detected in vitro and in vivo. Functional studies will be necessary to determine the precise biological impact of these variants.

Despite the utility of in vitro models, none of the marine strains reached intracellular replication levels comparable to those of *B. abortus* 2308W. This variability is consistent with previous reports showing divergent intracellular behaviors among marine and ST27 *Brucella* strains in epithelial and macrophage cells [51,52]. Differences in host cell type, immune response, and intracellular trafficking strategies likely contribute to these discrepancies [53,54,55]. A comprehensive evaluation of intracellular virulence would benefit from the use of multiple cell types and from the assessment of canonical *Brucella* virulence regulators, such as BvrR/BvrS, VjbR, VirB, and MucR [34,44,56,57,58].

In vivo, both *K. sima* isolates persisted in the mouse spleen, with lower bacterial burdens and milder pathological scores than in *B. abortus* 2308W, closely resembling marine reference strains. These findings align with previous murine studies reporting attenuation of *B. pinnipedialis* and *B. ceti* strains in spleen and liver tissues [9,44]. Notably, although bmarCR39b and bmarCR42b achieved substantial early splenic colonization, they induced limited hematological alterations and pathology in mice. Such persistence with reduced pathology has not been previously described for ST27 strains. Although the ST27 isolates displayed lower bacterial burdens and milder histopathological alterations compared to *B. abortus* 2308W in the murine model, these findings do not indicate enhanced virulence relative to the terrestrial reference strain. Importantly, virulence in *Brucella* spp. is strongly host-dependent, and attenuation observed in experimental murine systems does not necessarily exclude significant pathogenic potential in natural cetacean hosts or in humans. Therefore, the intermediate phenotype observed in mice should be interpreted within the biological context and host specificity of marine *Brucella* strains.

Overall, our study provides the first comprehensive characterization of pathogenic traits in ST27 *Brucella* isolates from *K. sima*, highlighting both their virulence-associated features and the limitations of current models in predicting disease severity across hosts. From a One Health perspective, these findings emphasize that the pathogenicity of marine *Brucella* strains is tightly linked to host-specific adaptations that conventional experimental systems may not adequately capture. As demonstrated for other host-adapted *Brucella* species, refining or developing alternative models may be necessary to assess better virulence, transmission risk, and zoonotic potential [59].

Future studies should evaluate ST27 pathogenicity in additional cellular systems, including macrophages and, when feasible, cetacean-derived cells, to better reflect natural host conditions. Functional characterization of isolate-specific genomic variants and targeted analysis of canonical virulence regulators, such as BvrR/BvrS and the VirB type IV secretion system, will be essential to clarify the molecular basis of the strain-dependent phenotypes observed. Refinement of host-adapted experimental models will also be necessary to improve zoonotic risk assessment and reconcile experimental attenuation with natural disease severity.

## Figures and Tables

**Table 1:** *Brucella* strains included in this study.

Strain	Characteristics	Source/Reference
*B. abortus* (2308W)	Wild-type, virulent, biotype 1, Nalr spontaneous mutant of strain 2308	[23]
*B. canis* (bcanCR12)	Wild-type, virulent	[24]
*B. abortus* (2.13)	2308 Nalr bvrS::Tn5	[25]
*B. ceti* (B1/94)	Wild-type, virulent, porpoise type	[8]
*B. ceti* (B14/94)	Wild-type, virulent, dolphin type	[8]
*B. pinnipedialis* (B2/94)	Wild-type, virulent, seal type	[8]
*Brucella* sp. (bmarCR39b)	Wild-type, virulent, isolated from mother	[18]
*Brucella* sp. (bmarCR42b)	Wild-type, virulent, isolated from fetus	[18]

**Table 2:** Hematological values of CD1 mice after 7 days post-injection with 10^6^ CFU.

Parameter	Unit	Mon-Infected Mice	*B. abortus* (2308)	*B. ceti* (B14/94)	*B. pinnipedialis* (B2/94)	*Brucella* sp. (bmarCR39b)	*Brucella* sp. (bmarCR42b)
		Mean	Mean	Mean	Mean	Mean	Mean
Leucocytes	10^9^/L	10.6	5.6	7.3	8.1	5.1	10.3
Lymphocytes	10^9^/L	8.3	3.4	5.6	6.4	3.5	7.1
Monocytes	10^9^/L	0.2	0.3	0.3	0.2	0.2	0.2
Neutrophils	10^9^/L	1.6	1.9	1.4	1.5	1.3	3.0
Lymphocytes	%	80.4	62.0	76.9 (*)	77.5 (*)	69.8	68.8
Monocytes	%	2.3	6.3	3.6	2.1	4.7	2.1
Neutrophils	%	15.1	31.7	19.5 (*)	20.4	25.5	29.1
Red blood cells	10^12^/L	11.2	6.8	7.9	8.5	7.5	8.4
Hemoglobin	g/dL	17.0	10.7	12.6	13.1	10.8	13.3
Hematocrit	%	64.0	37.6	44.3	48.1	42.5	45.1
Platelets	10^9^/L	-	613.2	382.5 (*)	455.2	716.2	497.8

Values are presented as mean ± SD. An asterisk (*) indicates a statistically significant difference compared with non-infected control mice (*p* ≤ 0.05). Statistical comparisons were performed using one-way ANOVA followed by Tukey’s multiple comparison test.

**Table 3:** Hematological values of CD1 mice after 30 days post-injection with 10^6^ CFU.

Parameter	Unit	Non-Infected Mice	*B. abortus* (2308)	*B. ceti* (B14/94)	*B. pinnipedialis* (B2/94)	*Brucella* sp. (bmarCR39b)	*Brucella* sp. (bmarCR42b)
		Mean	Mean	Mean	Mean	Mean	Mean
Leucocytes	10^9^/L	10.6	8.2	9.6	9.5	7.4	7.4
Lymphocytes	10^9^/L	8.3	3.8	7.9 (*)	7.7	5.8	5.5
Monocytes	10^9^/L	0.2	0.3	0.4	0.2	0.2	0.2
Neutrophils	10^9^/L	1.6	3.3	1.4 (*)	1.8	1.4 (*)	1.6 (*)
Lymphocytes	%	80.4	50.2	81.8 (*)	79.2 (*)	79.6 (*)	77.2
Monocytes	%	2.3	4.2	4.3	2.0	2.6	2.6
Neutrophils	%	15.1	46.7	13.9 (*)	18.8	17.8	20.2
Red blood cells	10^12^/L	11.2	9.2	9.2	9.0	8.2	9.5
Hemoglobin	g/dL	17.0	12.2	14.1	13.8	12.1	13.8
Hematocrit	%	64.0	42.0	50.3	48.0	42.6	47.6
Platelets	10^9^/L	-	531.5	495.5	395.5	395.8	436.8

Values are presented as mean ± SD. An asterisk (*) indicates a statistically significant difference compared with non-infected control mice (*p* ≤ 0.05). Statistical comparisons were performed using one-way ANOVA followed by Tukey’s multiple comparison test.

## Data Availability

The original contributions presented in this study are included in the article/Supplementary Material. Further inquiries can be directed to the corresponding author.

## Supplementary Materials

The following supporting information can be downloaded at: https://www.mdpi.com/article/10.3390/tropicalmed11040098/s1, Dataset S1: De novo assembly summary statistics and genome-wide variants identified by reference-based mapping against *Brucella suis* 1330, including SNPs, indels, and complex mutations in coding and intergenic regions.

## Abbreviations

The following abbreviations are used in this manuscript:

ANOVA	Analysis of variance
ATCC	American Type Culture Collection
AO	Alternate allele read count
BWA-MEM	Burrows–Wheeler Aligner (Maximal Exact Matches)
CFU	Colony-forming units
CO_2_	Carbon dioxide
CICUA	Comité Institucional para el Cuidado y Uso de los Animales
DMEM	Dulbecco’s Modified Eagle Medium
DP	Total read depth
HRP	Horseradish peroxidase
IgG	Immunoglobulin G
indels	Insertions and deletions
LPS	Lipopolysaccharide
MAG	Ministry of Agriculture (Costa Rica)
MIC	Minimum inhibitory concentration
MLST	Multilocus sequence typing
MOI	Multiplicity of infection
PBS	Phosphate-buffered saline
PVDF	Polyvinylidene difluoride
QUAL	Phred-scaled quality score
RO	Reference allele reads
rpm	Revolutions per minute
SDS-PAGE	Sodium dodecyl sulfate–polyacrylamide gel electrophoresis
SENASA	Servicio Nacional de Salud Animal
SNP	Single-nucleotide polymorphism
ST27	Sequence type 27
TSA	Trypticase soy agar
TSB	Trypticase soy broth
WGS	Whole-genome sequencing
